# Integrated thermal emission microchip based on meta-cavity array

**DOI:** 10.1515/nanoph-2022-0328

**Published:** 2022-08-11

**Authors:** Qiongqiong Chu, Fengyuan Zhang, Ye Zhang, Tong Qiao, Shining Zhu, Hui Liu

**Affiliations:** National Laboratory of Solid State Microstructures, School of Physics, Collaborative Innovation Center of Advanced Microstructures, Nanjing University, Nanjing, Jiangsu 210093, China

**Keywords:** meta-cavity, metasurfaces, optical absorption, thermal emission

## Abstract

Microscale infrared thermal emitters are highly demanded in a variety of applications such as micro-molecular thermal sensing and micro-thermal imaging. In this paper, we propose a micro-meta-cavity array through combining nanohole metasurfaces and Fabry–Pérot (FP) cavity. Based on this design, integrated multiband micro-thermal emitters covering 7 − 9 μm and 10 − 14 μm wavelength ranges with high spatial resolution near wavelength scale has been theoretically and experimentally demonstrated simultaneously, providing the possibility for microscale infrared sources. In addition, narrow thermal emission bandwidth is enabled by the interaction between the resonant modes of metasurface and the FP cavity mode in meta-cavity. The emission features of each meta-cavity are investigated and analyzed through thermal imaging. Furthermore, polarization, wavelength and spatial multiplexing thermal emission with high spatial resolution is also experimentally demonstrated utilizing nanohole patterns. We anticipate that this thermal emission microchip can be possibly employed in micro-molecular sensing and micro-thermal imaging in the future.

## 1 Introduction

Recently, infrared selective thermal emitters have been proposed to act as cost-efficient infrared sources, providing a new platform for plenty of infrared applications in biochemical sensing [1, 2], thermal imaging [3–6], thermophotovoltaics [7, 8], radiative cooling [9–12], super-Planckian near-field thermal radiation [13], and spectroscopy characterization [14, 15]. Based on a variety of optical designs, such as grating [16–20], FP cavity [21–23], Tamm plasmon polaritons (TPPs) [24–26], photonic crystals [27, 28], metamaterials [29–31], metallic [32–39] and dielectric metasurfaces [40–43], flexibly controllable thermal emitters have been proposed.

With the rapid development of infrared applications, there is an increasing demand for micro-molecular sensors with multiple characteristic wavelengths in LWIR (long-wave infrared range). It is of great significance to study the multiband thermal emitters which can cover the LWIR as much as possible. The research of thermal emitters with high spatial resolution is also crucial for the applications of infrared micro-molecular sensing and imaging. Although plasmonic metasurfaces based multiband thermal emitters have been proposed, exposed dielectric middle spacers with infrared emission in these structures would increase the background thermal emission and then subsequently weaken the contrast between desirable thermal emission signal and background noise in thermal imaging. Higher thermal imaging contrast is beneficial for achieving higher spatial resolution. A microchip which simultaneously possesses multiband thermal emission peaks in LWIR and highly improved thermal imaging contrast is rarely reported up to now.

In this work, based on a micro-meta-cavity array that combines metasurfaces and FP cavity, we propose a thermal emission microchip with high spatial resolution. Various meta-cavities can be designed for desired resonant wavelengths. We have designed a 3 × 3 meta-cavity array to realize multiband thermal emission by varying the length and period of the nanohole unit cell, covering two LWIR domains 7 − 9 μm and10 − 14 μm. The coupling between metasurfaces and FP cavity is manipulated to realize narrower thermal emission peak than that of a sole metasurface or FP cavity. Through designed meta-cavity patterns, we experimentally demonstrate polarization, wavelength and spatial multiplexing thermal emission with a high spatial resolution near wavelength scale. The high spatial resolution obtained is attributed to the large thermal imaging contrast between the meta-cavity and the surrounding planar gold layer. This meta-cavity design opens up a new avenue for micro-thermal imaging and micro-molecular sensing.

## 2 Results and discussion

### 2.1 Design of meta-cavity array

**Figure 1:**
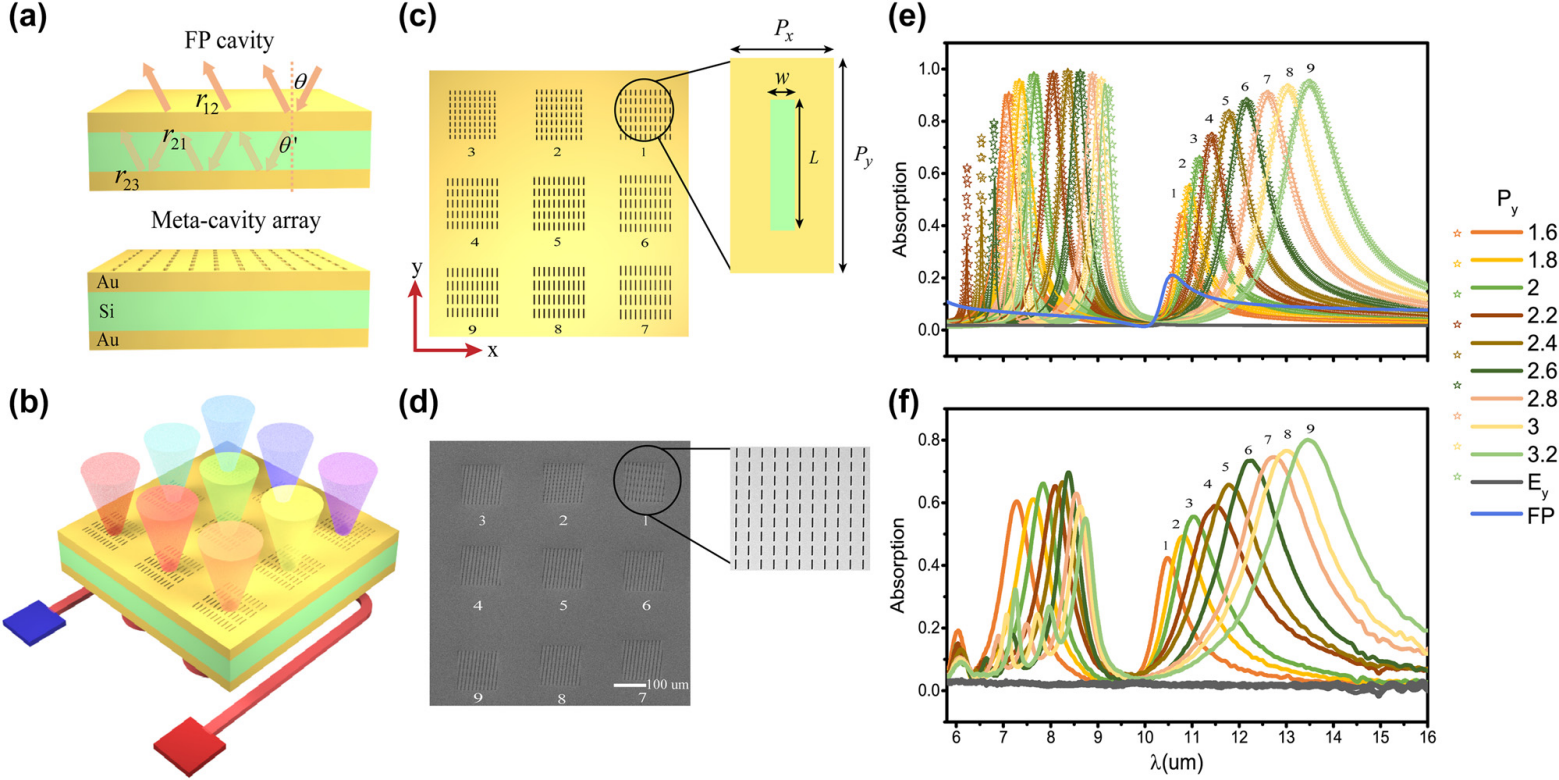
Meta-cavity array based multiband microchip. (a) Sketch of the conventional FP cavity and meta-cavity. (b) Sketch of designed multiband microchip. The color of the emitted light from violet to red represents the wavelength of thermal emission that changes from short-wave to long-wave domain. Designed (c) and fabricated (d) meta-cavity array on the top view. The insets of (c) and (d) depicts the unit cell of meta-cavity array and zoomed-in SEM image of fabricated first meta-cavity. (e) Absorption spectra of meta-cavity array simulated (solid lines) and calculated (marked lines) under x-polarized incidence. The gray lines present the simulated absorption spectra of meta-cavity array under y-polarized incidence. The blue line presents the simulated absorption spectrum of individual FP cavity. (f) Measured absorption spectra of meta-cavity array.

Conventionally, FP cavity mode can be regarded as a standing wave formed by the interference of multiple reflected lights. In the process, the sum of the reflection phase from the cavity interfaces and the propagation phase inside the cavity medium should satisfy the condition *φ* = 2*m*π(*m* = 1, 2, 3…). This means that the resonance of a cavity is mainly determined by the phase accumulation of light in one oscillation period. Therefore, we can control the resonance mode through tailoring the accumulated phase of light oscillating inside the cavity. As shown in Figure 1(a), the electromagnetic properties of conventional FP cavity are frequently analyzed by interference theory [44, 45], according to which, the reflection of FP cavity can be directly described as:

(1) $$R={\left|r_{12}+\frac{t_{12}t_{21}r_{23}{\text{ e}}^{\left(-2i\varphi \right)}}{1-r_{21}r_{23}{\text{ e}}^{\left(-2i\varphi \right)}}\right|}^{2}$$

where *φ* = *nkd* cos *θ*′ represents the propagation phase, *d* and *n* are the thickness and refractive index of the middle spacer, *k* is the free space wavenumber. We set *θ*′ as 0 for normal incidence. Here, *r*_12_(*t*_12_) and *r*_21_(*t*_21_) are the reflection (transmission) coefficients of the top reflection interface between different materials while *r*_23_ presents the reflection coefficient of the bottom reflection interface. Correspondingly, *φ*_*r*12_, *φ*_*r*21_, *φ*_*r*23_, *φ*_*t*12_ and *φ*_*t*21_ are defined as the phase of the reflection and transmission coefficients. Since the transmission is zero, the absorption (A) of FP cavity can be calculated as *A* = 1 − *R*. Here, if the top and bottom reflection interfaces are the same, we will have $\left|r_{12}\right|$ ≈ $\left|r_{21}\right|=\left|r_{23}\right|$ , which means that the only way to manipulate resonant wavelengths of pure FP mode is to change the thickness of the middle spacer.

Metasurfaces [46–50], characterized by two-dimensional subwavelength nanostructures, have been demonstrated to manipulate the properties of electromagnetic waves, such as amplitude, phase, and polarization. By replacing the top reflection interface with metasurface, we propose a new kind of FP cavity called as meta-cavity which provides more degrees of freedom to control the resonant modes by artificially modifying *r*_12_(*t*_12_) and *r*_21_(*t*_21_). Linearly tunable wavelengths over a wide range can be achieved through continuously adjusting the structural parameters of metasurfaces. Here, we demonstrate a meta-cavity array composed of 3 × 3 nanohole metasurfaces, a gold mirror, and a dielectric Si layer sandwiched between them, to form a functional microchip possessing multiple resonant wavelengths, as schematically illustrated in Figure 1(b). Each metasurface is formed by artificially designed unit cell to obtain desired resonant wavelengths. In particular, the unit cell period along *x* axis and the width of nanohole are fixed as Px = 1.56 μm, *w* = 0.2 μm. The length of the nanohole depends on the unit cell period along *y* axis Py and is defined as *L* = 0.7 × Py. We change Py from 1.6 μm to 3.2 μm with a step of 0.2 μm to form nine different metasurfaces. The top view of designed meta-cavity array is shown in Figure 1(c).

The electromagnetic properties of individual metasurface are firstly analyzed. As the polarized electric field of the nanohole is usually perpendicular to its long axis, each nanohole can be regarded as an x-polarized electric dipole. Numerical simulations were performed to calculate the transmission and reflection coefficients for each individual metasurface and single Au mirror under x-polarized incidence. The results are given in Supporting Information, Figures S1 and S2. We observe that each metasurface exhibits two resonant modes, providing two phase changes and amplitude variations. Also, it should be noted that when the incident light is y-polarized, the resonant modes of nanoholes cannot be excited, and then those metasurfaces will subsequently be equivalent to a planar gold slab.

Based on abrupt phase and amplitude variations induced by metasurfaces, the total absorption of meta-cavity array can be calculated (marked lines) based on Eq. (1), as shown in Figure 1(e). In order to further confirm the accuracy of calculated results, we also simulated the absorption spectra (solid lines) of meta-cavity array, as shown in Figure 1(e). For comparison, individual FP cavity mode was simulated by replacing metasurfaces with a planar gold slab with thickness of 10 nm. It is clear that the theoretically calculated results agree well with the simulated results. Each metasurface of meta-cavity brings two abrupt phase changes that, respectively, shift the FP mode to two bands, 7 − 9 μm and 10 − 14 μm, manifesting as two absorption peaks within LWIR domain (see details in Supporting Information, Figure S3). As Py and *L* increase, two absorption peaks of meta-cavity gradually shift to long-wave range along with the enhancement of absorption amplitude. Benefiting from the interaction between metasurface and FP cavity, the closer the resonance wavelength is to the individual FP cavity mode, the higher the quality factor of the absorption peak will be obtained. Additionally, when Py increases to 2.2 μm, a third absorption peak will appear in short-wave domain. Since these third peaks with low absorption of meta-cavities are mostly located in atmospheric absorption window, no further analysis is performed here.

### 2.2 Multiband thermal emission microchip

In an effort to experimentally validate the infrared performance of proposed microchip, designed 3 × 3 metasurface array was fabricated on the top gold layer. Figure 1(d) illustrates a scanning electron microscopic (SEM) top view image of the fabricated meta-cavity array. The absorption spectra measured with a Fourier transform infrared (FTIR) spectrometer at room temperature are presented in Figure 1(f). We can see that measured absorption spectra are consistent with the simulated results. Similarly, when Py and *L* increase, linearly tunable resonant peaks with improved *Q* factors are experimentally realized in LWIR domain, exhibiting advantages as multi-resonant microchip. Specifically, measured results and simulated results match well within 10 − 14 μm while there are slight discrepancies within 7 − 9 μm. These discrepancies might be attributed to the small difference between the actual refractive index of Si and the value set in the simulation process. In addition, the *Q* factors of experimental results are slightly reduced compared to simulation results due to fabrication errors.

According to Kirchhoff’s Law, the thermal emissivity of meta-cavity is regarded as the absorptivity at thermal equilibrium. Therefore, we can calculate the thermal emissivity of designed meta-cavity array through its absorption profiles discussed above. Here, we utilize thermal camera to investigate the thermal emission properties of meta-cavities. The thermal emission intensity is denoted with the measured temperature in the thermal images. The temperature rendered in thermal images is not equal to the absolute temperature of the sample, so we will call it emission temperature in the following.

**Figure 2:**
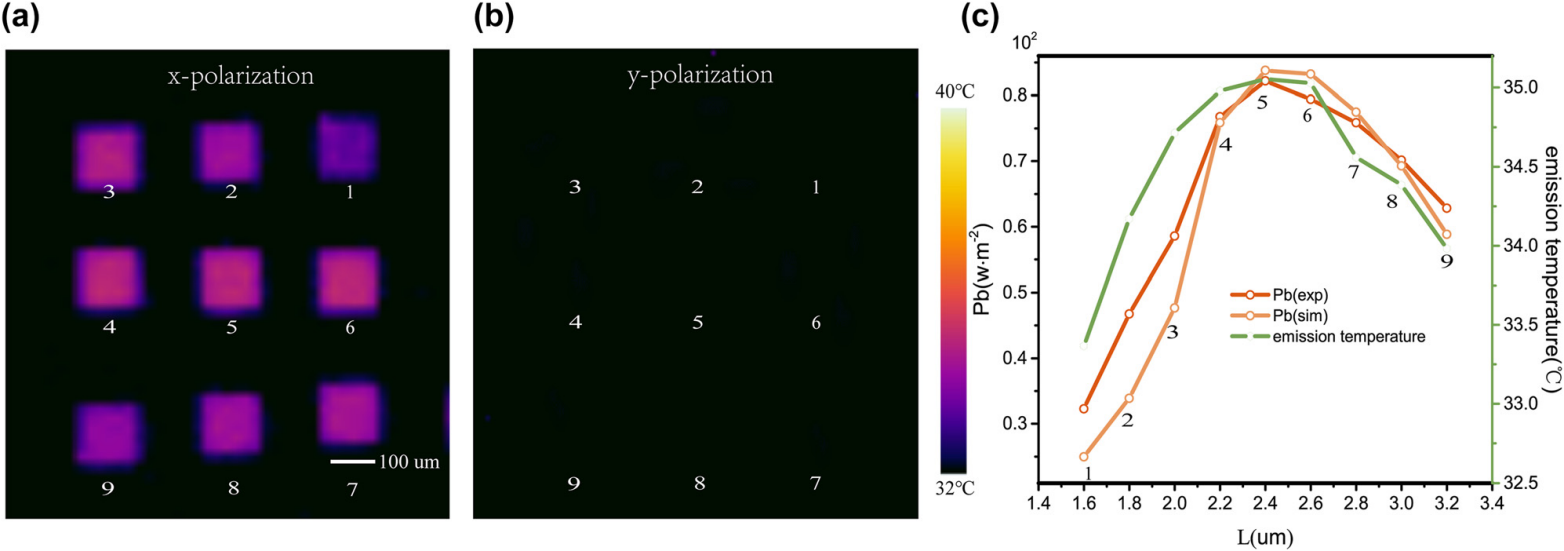
Thermal images of meta-cavity array based thermal emission microchip. The thermal images taken under x polarizer (a) and y polarizer (b) after heating the sample to 100 °C. (c) Calculated thermal radiation energy Pb_exp_, Pb_sim_ and the average emission temperature of each meta-cavity structure under x polarizer.

In experiments, a polarizer was put between the meta-cavity sample and the thermal camera. After heating the sample to 100 °C, thermal images were taken under two orthogonal polarizations, as shown in Figure 2(a) and (b). The polarization of detected thermal emission from the sample can be continuously changed through rotating the polarizer from 0 to 2*π*. Under x-polarization filtering, each meta-cavity shows higher temperature than background area around it, indicating their strong x-polarized thermal emission. However, in Figure 2(b), the thermal emission under y polarization is very weak. With different thermal emission intensities, nine meta-cavities subsequently render different emission temperatures in the thermal image, demonstrating the realization of integrating multiple micro-thermal emitters in one microchip. So far, we have experimentally and theoretically verified the feasibility of designed thermal emission microchip for thermal emissivity manipulation within LWIR domain. Moreover, compared to reported optical multi-resonant chips, our designed laser-free thermal emission microchip can greatly reduce the operating costs, making it suitable for potential applications.

We know that the thermal radiation energy from heated blackbody can be characterized by the Stefan–Boltzmann Law, *P* = *ɛσT*^4^ where *ε* and *T* are the emissivity and temperature of sample and *σ* is the Stefan–Boltzmann constant. The thermal camera used in our experiments obtains the thermal images by measuring the thermal emission (8 − 14 μm) according to Stefan–Boltzmann law and the internal algorithm of corresponding software. To better understand the relationship between thermal images and thermal emissivity of each meta-cavity structure, we calculated the thermal radiation energy of each meta-cavity, Pb_exp_ and Pb_sim_, based on experimental and simulated emissivity respectively (see details in Supporting Information). Then Pb_exp_, Pb_sim_ and the corresponding average emission temperature of each meta-cavity are simultaneously plotted in Figure 2(c). It is clear that the thermal radiation energy is proportional to the emission temperature of thermal images. Although thermal radiation energy (Pb_exp_ and Pb_sim_) shows slight discrepancies with average emission temperature, three curves share the same trend as Py and *L* increase, which proves that thermal images can directly demonstrate the emission intensities of meta-cavities.

### 2.3 Multiplexing thermal emission with high spatial resolution

**Figure 3:**
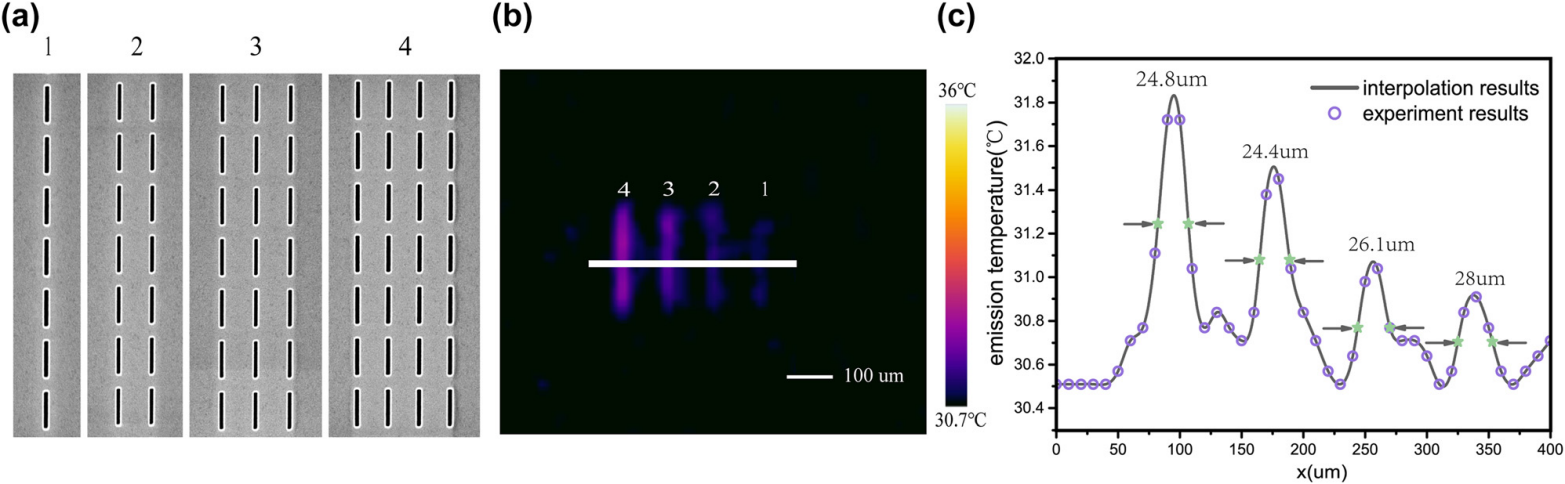
Spatially designed thermal emission based on wire-shaped meta-cavity patterns. (a) SEM top view of fabricated wire-shaped meta-cavity patterns. (b) The thermal image of fabricated patterns taken at 100 °C. (c) The emission temperature distribution at the center of four samples. The purple circle presents the emission temperature directly obtained from thermal image. The gray solid line presents the emission temperature distribution obtained by interpolation.

In above, our results show that thermal emission wavelength and intensity can be tuned through designed meta-cavity. In the following, we will show that the spatial profile of thermal emission can also be controlled through designed meta-cavity pattern of nanoholes. The emission information can be encoded into designed patterns. Firstly, four different meta-cavity patterns composed of 1, 2, 3, and 4 columns of nanoholes (Py = 1.8 μm) were designed and fabricated, as shown in Figure 3(a). Corresponding thermal image taken at 100 °C is illustrated in Figure 3(b). We observe that samples containing 3, 4 columns of nanoholes are clearly visible in the thermal image, whereas samples containing 1, 2 columns of nanoholes are not clear enough to be distinguished. This is because fewer columns of nanoholes will produce weaker thermal emission, resulting in unclear imaging, as given in Figure 3(b). Based on the emission temperature (purple circle) obtained from the thermal image, each linewidth of the emission temperature peaks was obtained by interpolation (solid gray line), as shown in Figure 3(c). We observe that a linewidth of 24.4 μm can be achieved when the sample is composed of 3 columns of nanoholes. This high spatial resolution is obtained from high thermal imaging contrast through suppressing the background emission temperature. By integrating designed micro-meta-cavity components, this thermal emission microchip can facilitate compact integrated high-resolution infrared micro-thermal emitters. In our experiments, the spatial resolution of thermal imaging is limited by the spatial resolution (10 μm) of the thermal camera used. If we use a thermal imaging camera with higher resolution in the future, we can further obtain higher spatial resolution of thermal emission.

**Figure 4:**
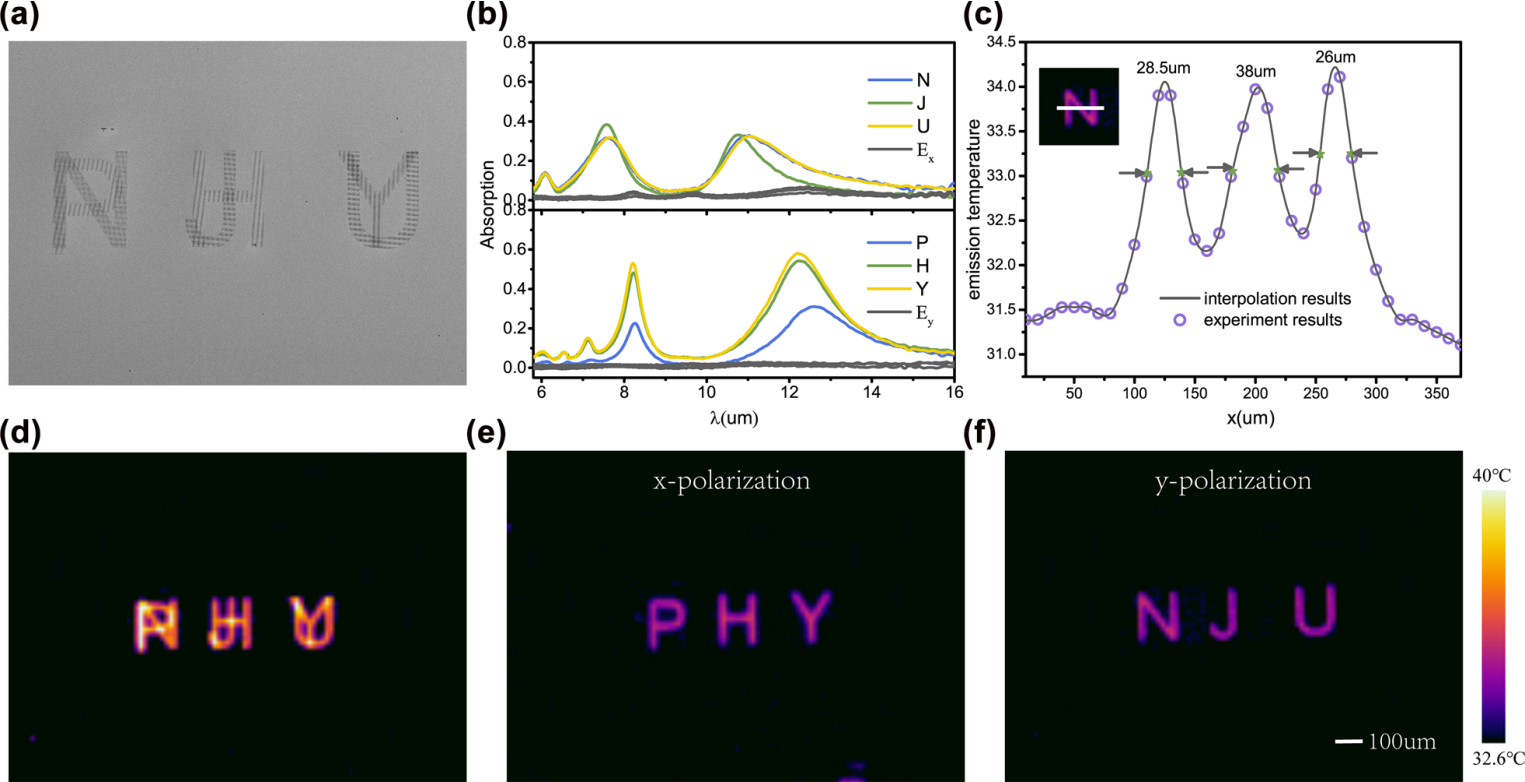
Multiplexing thermal emission based on complex meta-cavity patterns. (a) SEM top view of fabricated meta-cavity patterns. (b) Measured absorption spectra of the NJU (PHY) patterns under y-polarized and x-polarized incidence. (c) The emission temperature distribution along the center line of letter N. Thermal images of two fabricated patterns taken at 100 °C without polarizer (d), with x polarizer (e) and with y polarizer (f).

In above experiments, each meta-cavity pattern is separately distributed and characterized by single-channel thermal emission under x polarization selection. More complex meta-cavity patterns can be designed to achieve polarization, wavelength and spatial three-channel multiplexed thermal emission by controlling nanohole patterns. In experiments, two meta-cavity patterns were designed and fabricated for two polarizations, namely “NJU” and “PHY”, as illustrated in Figure 4(a). Two kinds of nanohole unit cells of Py = 1.8 μm and Py = 2.6 μm are chosen to form “NJU” and “PHY” respectively. In the patterns, we set the long axis of the nanohole arrays along the *x* and *y* axis directions respectively. To experimentally investigate the multiplexed thermal emission phenomenon of two fabricated patterns, the absorption spectra of each letter under two polarizations were measured, as shown in Figure 4(b). It can be seen that under the excitation of x-polarized light, absorption peaks appear in the PHY pattern while the absorption is almost zero under y-polarized incidence. On the contrary, under the excitation of y-polarized incidence, the NJU patterns show absorption peaks while the absorption is almost zero under x-polarized incidence. Theoretically, the absorption spectrum of each letter of NJU (PHY) should be the same as the absorption spectrum of periodic meta-cavity structure with a unit cell of Py = 1.8 μm (Py = 2.6 μm). However, there are slight differences between N, J, and U due to different selected areas in FTIR spectrometer measuring process. Under the same measurement environment, the absorption (emissivity) of the letter P is smaller than that of the letters H and Y. This is because the emission area of the letter P is smaller, resulting in a weaker emission signal than other letters. Besides, the difference in the linewidth of the absorption peaks of N, U and J is caused by fabrication errors in experiments.

To intuitively display the multiplexing thermal emission phenomenon, three thermal images were separately taken under the condition of without polarizer, with x polarizer and with y polarizer, as shown in Figure 4(d)–(f). Two independent information encoded into a single meta-cavity sample through two polarization channels can be decoded by rotating the polarizer before the thermal camera. When the polarizer is not added, the thermal emissions from both patterns are received and superimposed in the same overlap region. When the x(y) polarizer is added, only the “PHY” (“NJU”) pattern is visible in the thermal image. To explore the spatial resolution of the thermal images, we selected the letter N and measured the intensity profile along a line through the letter, as shown in Figure 4(c). From the curve, we observe that a spatial resolution of approximate 30 μm can be achieved on the meta-cavity pattern. In addition, we can make a further outlook that if we add a suitable color filter between the sample and the thermal camera, it is feasible to achieve wavelength, polarization and spatial multiplexing thermal imaging at the same time. Although the wavelength selectivity is not reflected in the thermal imaging experiments, we can reasonably infer that this scheme is indeed feasible based on the absorption spectra of two fabricated patterns shown in Figure 4(b).

Above emission results are analyzed at normal direction. The thermal emission properties of meta-cavity patterns at off-normal direction have also been investigated from both experiment and simulation results (see details in Supporting Information, Figures S4 and S5). The results show that the meta-cavity design has low dependence on the angle. We can still obtain stable thermal emission properties even at very oblique angles. This wide-angle thermal emission characteristic makes our design promising for micro-thermal imaging and micro-thermal sensing.

## 3 Conclusions

In summary, based on micro-meta-cavity array, we have proposed a thermal emission microchip to realize multiband thermal emission covering two LWIR domains 7 − 9 μm and 10 − 14 μm with high spatial resolution near-wavelength scale. By utilizing artificially designed metasurfaces, required thermal emission wavelengths can be obtained. Except for meta-cavity array, we further investigate meta-cavity patterns ranging from simple wire-shaped patterns to complex letter patterns and demonstrate polarization, wavelength and spatial multiplexing high-resolution thermal emission.

Our results imply that designed microchip is robust over a large infrared range for wide-angle thermal emissivity manipulation. The experimentally demonstrated properties of high spatial resolution and multiplexing capability can greatly increase the infrared information storage capacity of the meta-cavity design, making it a promising candidate for compact infrared applications, such as micro-molecular fingerprints detection. By integrating micro-meta-cavity components, this design can be expected to obtain the spectral fingerprints through thermal image pixels, providing a new method for molecular sensors. Furthermore, without the need for expensive infrared source, proposed laser-free thermal emission microchip is low-cost, making it more appealing for mentioned potential applications.

## 4 Methods

### 4.1 Thermal emission microchip fabrication

Firstly, three layers of Au (70 nm)/Si(1.36 μm)/Au(100 nm) are sequentially deposited on the Si substrate by magnetron sputtering (ULVAC CS-200z). Then 3 × 3 nanohole metasurfaces are etched from the top gold film by a focused ion beam (FIB dual-beam FEI Helios 600i, 30 keV, 100 pA). By adjusting the etching parameters, the length of nanohole L can be varied from 1.12 μm to 2.24 μm while the etching depth is set as 70 nm. Each metasurface of meta-cavity array is 120 μm × 120 μm sized.

### 4.2 Optical characterization

Polarized reflection spectra (R) of the meta-cavity array were measured in the spectral range of 3 − 18 μm using a Fourier transform infrared (FTIR) spectrometer. The reflection signals were collected using a Hyperion 2000 IR microscope with a liquid-nitrogen-cooled HgCdTe (MCT) detector. Measured reflection spectra were normalized with respect to a gold mirror. The absorption spectra (A) can be derived on the basis of the measured reflection spectra (R) by *A* = 1 − *R*.

### 4.3 Infrared thermal imaging

The sample is heated to 100 °C using a Linkam THMSG600 thermal stage and then the resulting thermal emission within 8 − 14 μm is detected by a thermal camera (Fotric 288) and characterized by infrared thermal images.

### 4.4 Numerical simulations and analysis

Numerical simulations are performed with 3D finite difference time domain method (FDTD) to calculate the transmission and reflection coefficients for each individual metasurface and single Au mirror under x-polarized incidence, as shown in Figures S1 and S2. Periodic boundary conditions are applied in both *x* and *y* directions for mimicking the periodic nanohole array. The absorption spectra of meta-cavity array are simulated under the same condition.

## Supplementary Material

Supplementary Material Details
