# Supplementary material for: Integrated thermal emission microchip based on meta-cavity array
Source: Nanophotonics. 2022 Aug 11;11(18):4263–71. doi: 10.1515/nanoph-2022-0328 (PMC11501492; doi:10.1515/nanoph-2022-0328)
Supplement: Supplementary file 1 — Supplementary Material Details [file j_nanoph-2022-0328_suppl.docx]

Supporting Information

**Integrated Thermal Emission Microchip Based On Meta-Cavity Array**

Qiongqiong Chu ^1^, Fengyuan Zhang ^1^, Ye Zhang ^1^, Tong Qiao ^1^, Shining Zhu ^1^ and Hui Liu^1, *^

^1^National Laboratory of Solid State Microstructures, School of Physics, Collaborative Innovation Center of Advanced Microstructures, Nanjing University, Nanjing, Jiangsu 210093, China.

1. **Reflection coefficients of metasurfaces**

In simulations, the transmission and reflection coefficients for each individual metasurface under x-polarized incidence were calculated, as shown in Figure S1 and S2. The transmission of AU mirror is out of consideration due to its opaque thickness. Because of $t_{12}=t_{21}$, we only present the phase and amplitude of $t_{12}$. Figure S1 displays the amplitude of simulated S parameters. It shows that when the incident light is x-polarized, two resonance modes can be excited in each metasurface and gradually shift to long-wave domain with increasing nanohole length. Next, the phase of simulated S parameters is investigated, as shown in Figure S2. Similarly, the change of phase shares the same shift tendency with the variation of amplitude as the length of nanohole increases. Multiple abrupt phase changes with Lorentz line shape are induced at resonant wavelengths. By artificially designing the metasurface, demanded phase change for target resonant wavelength can be obtained while the traditional reflection phase of planar Au layer is approximately fixed at π.


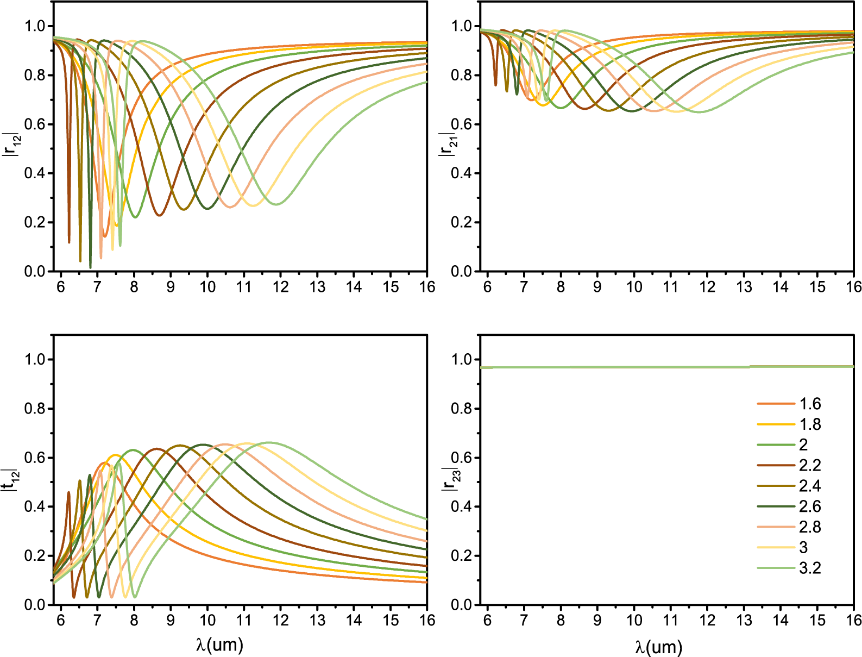


Figure S1. The amplitude of simulated S parameters. The amplitude of reflection coefficients $r_{12}$ (a) and $r_{21}$ (b) and transmission coefficient $t_{12}$ (c) for each individual metasurface under x-polarized incidence. (d) The amplitude of reflection coefficient $r_{23}$ of planar gold slab.


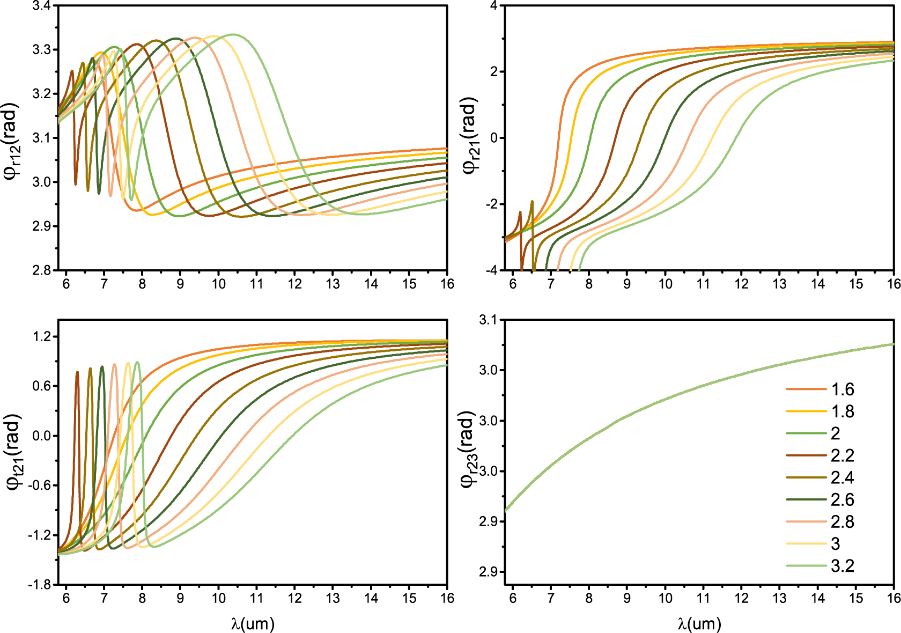


Figure S2. The phase of simulated S parameters. The phase of reflection coefficients $r_{12}$ (a) and $r_{21}$ (b) and transmission coefficient $t_{12}$ (c) for each individual metasurface under x-polarized incidence. (d) The phase of reflection coefficient $r_{23}$ of planar gold slab.

1. **Absorption properties of meta-cavities**

For an ordinary FP cavity with a spacer layer sandwiched between two gold slabs, the resonant modes satisfy the following condition:

${2\varphi}_{Au}+{2\varphi}_{p}=2m\pi$ ($m=1, 2\ldots$) (S1)

Here, $\varphi_{Au}$ is the reflection phase of planar gold slab and $\varphi_{p}$ is the propagation phase. Replacing the top reflection interface with metasurfaces, the accumulated phase $P_{total}$ of resonant modes can be tailored as:

$\varphi_{Au}+\varphi_{r21}+{2\varphi}_{p}=2m\pi$ ($m=1, 2\ldots$) (S2)

where, $\varphi_{r21}$ is the reflection phase of individual metasurface. The left-hand side of equation S2, $P_{total}$ of meta-cavity array and the right-hand side of equation S2, 2*m*π (*m*=1,2,3), are plotted together in Figure S3. The intersection points of them indicate the resonant wavelengths of tailored FP modes which are consistent with the resonant positions of absorption spectra.


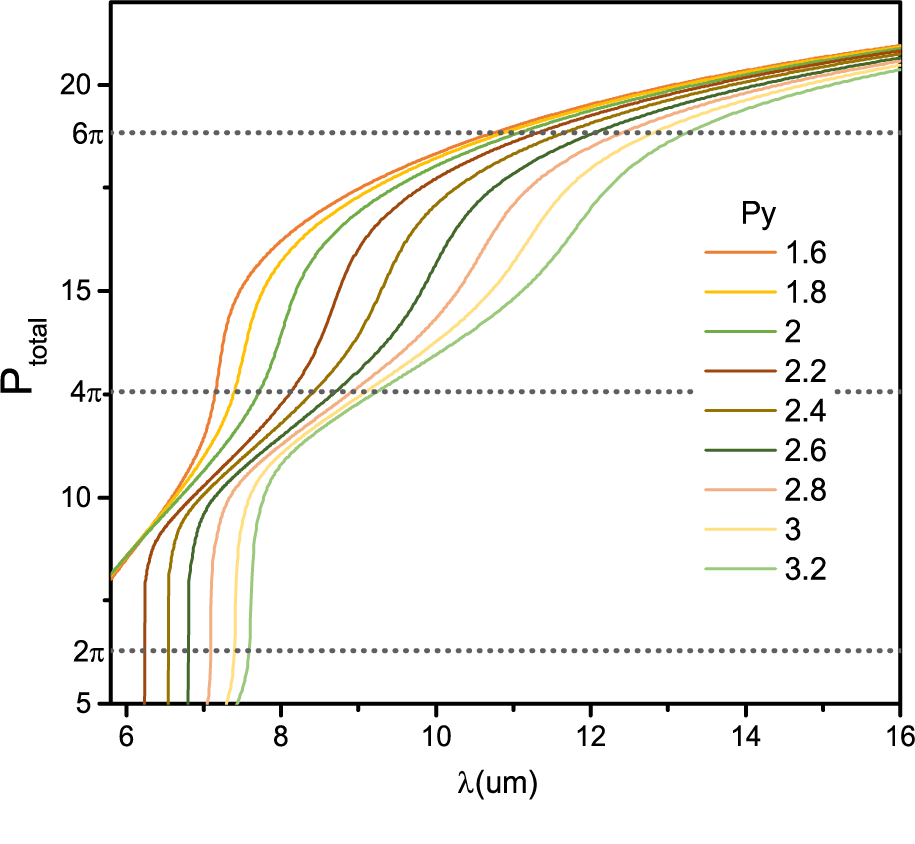


Figure S3. Accumulated phase inside meta-cavity array. The intersection points of phase variations and 2*m*π indicate the resonant wavelengths.

1. **Thermal emission of meta-cavities**

The thermal radiation energy of each meta-cavity within 8-14 um can be expressed by the following equation: ^[1,2]^

PB=$\int_{\lambda_{1}}^{\lambda_{2}} \varepsilon(\lambda)M_{bb}(\lambda,T)$ (S3)

Where $\varepsilon$ is the emissivity of meta-cavity ($\varepsilon_{exp}=\frac{\varepsilon_{sample}}{\varepsilon_{blackbody}}=\frac{A_{sample}}{A_{blackbody}}, \varepsilon_{sim}=\frac{A_{sim}}{A_{blackbody}}=A_{sim}$). In experiments, the absorption spectrum ($A_{sample}$) of meta-cavity sample and the absorption spectrum ($A_{blackbody}$) of the carbon powder (can be approximately seem as blackbody) sample were firstly measured and then the emissivity was calculated as $\varepsilon_{exp}=\frac{A_{sample}}{A_{blackbody}}$. In simulation, we set $A_{blackbody}=1$and then the emissivity $\varepsilon_{sim}$ was obtained by $\varepsilon_{sim}=\frac{A_{sim}}{A_{blackbody}}$. $M_{bb}$ is calculated blackbody emittance according to Planck's law. Two kinds of thermal radiation energy ${Pb}_{exp}$ and ${Pb}_{sim}$ are separately calculated based on $\varepsilon_{exp}$ and $\varepsilon_{sim}$. Three curves share the same trend, confirming the feasibility of the emissivity manipulation through meta-cavity array.

1. **Thermal emission properties of meta-cavities at off-normal direction**

To investigate the thermal emission properties of meta-cavities at the off-normal direction, we select two kinds of meta-cavities ($P_{y}=1.8 \mu m$ and $P_{y}=2.6 \mu m$) and calculate the absorption spectra of them under oblique incidence ($0^{o}-{80}^{o}$), as shown in Figure S4. It can be seen that the absorption peaks within 8-14um have a wide-angle characteristic and remain almost unchanged at small incident angles, which means that the thermal emission of meta-cavities can also maintain their thermal emission characteristics at a wide angle range.


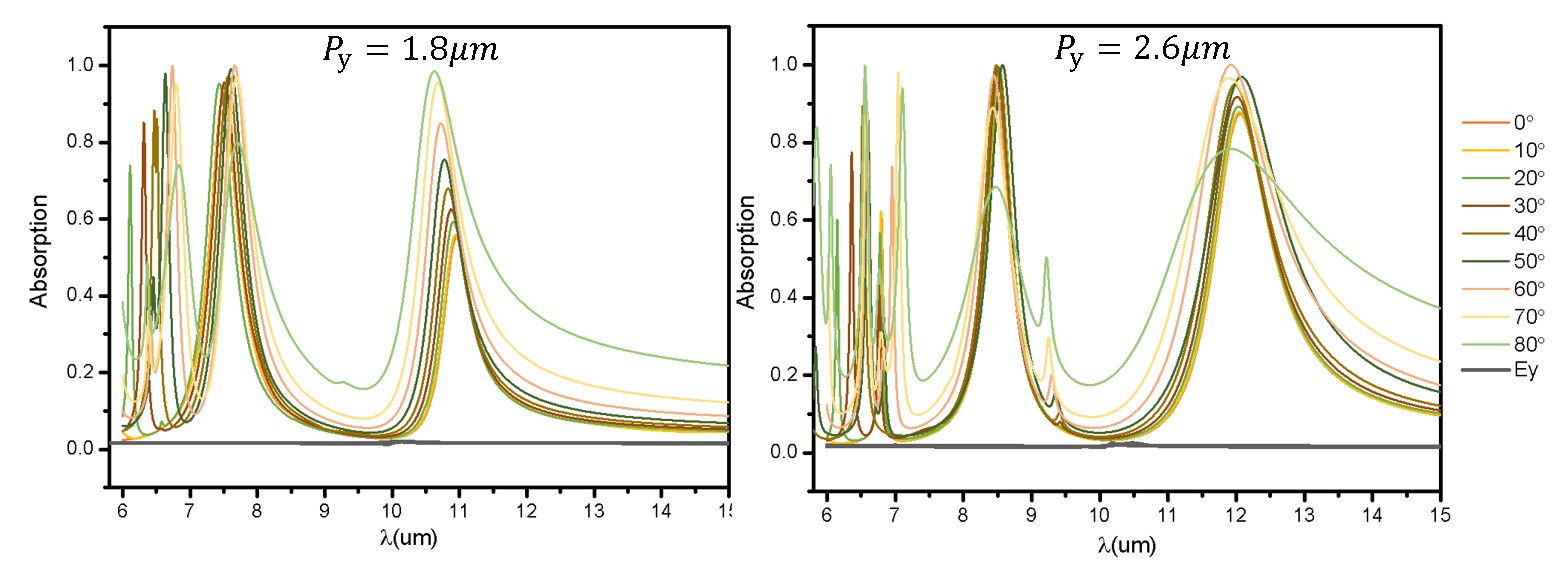


Figure S4. Absorption spectra of two kinds of meta-cavities under oblique incidence.

In experiments, we took the thermal images of the letters U ($P_{y}=1.8 \mu m$) and Y ($P_{y}=2.6 \mu m$) at the emission angle range of $0^{o}-{20}^{o}$ at a temperature of 100 ℃, as shown in Figure S5. We can see that the emission temperatures in the three images are basically the same, demonstrating that the thermal emission at small emission angles is stable, which is consistent with the theoretical calculation. Due to the limited imaging distance of the macro lens of our thermal camera, we can only take small-angle thermal images. However, we can reasonably infer that it is feasible to realize wide-angle thermal imaging (thermal emission) based on wide-angle absorption spectra.


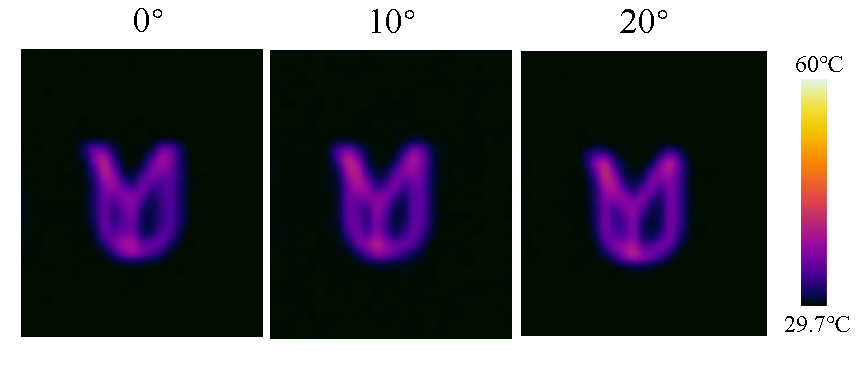


Figure S5. Thermal images of the letters U and Y taken at small angle.

**REFERENCES**

1. X. Y. Liu, W. J. Padilla, “Thermochromic Infrared Metamaterials,” Adv. Mater., vol. 28, no. 5, pp. 871-875, 2016.
2. J. Wang, F. B. Yang, L. J. Xu, J. P. Huang, “Omnithermal Restructurable Metasurfaces for Both Infrared-Light Illusion and Visible-Light Similarity,” Phys. Rev. Appl., vol. 14, no. 1, pp. 014008, 2020.
